# The prevalence of lymphatic filariasis infection and disease following six rounds of mass drug administration in Mandalay Region, Myanmar

**DOI:** 10.1371/journal.pntd.0006944

**Published:** 2018-11-12

**Authors:** Benjamin F. R. Dickson, Patricia M. Graves, Ni Ni Aye, Thet Wai Nwe, Tint Wai, San San Win, Myint Shwe, Janet Douglass, Richard S. Bradbury, William J. McBride

**Affiliations:** 1 College of Medicine & Dentistry, Division of Tropical Health and Medicine, James Cook University, Cairns, Queensland, Australia; 2 College of Public Health, Medical and Veterinary Sciences, Division of Tropical Health and Medicine, James Cook University, Cairns, Queensland, Australia; 3 James Cook University and World Health Organization Collaborating Centre for the Control of Lymphatic Filariasis, Soil Transmitted Helminths and Other Neglected Tropical Diseases, Cairns, Queensland, Australia; 4 Vector Borne Disease Control Unit, Ministry of Health and Sport, Naypyitaw, Myanmar; 5 Regional Vector Borne Disease Control Unit, Ministry of Health and Sport, Mandalay, Myanmar; 6 World Health Organization, Yangon, Myanmar; 7 General Practitioner, Mandalay, Myanmar; 8 Center for Neglected Tropical Diseases, Liverpool School of Tropical Medicine, Liverpool, United Kingdom; 9 School of Health, Medical and Applied Sciences, Central Queensland University, North Rockhampton, Queensland, Australia; Washington University School of Medicine, UNITED STATES

## Abstract

Lymphatic filariasis is widely endemic in Myanmar. Despite the establishment of an elimination program in 2000, knowledge of the remaining burden of disease relies predominantly on programmatic information. To assist the program, we conducted an independent cross-sectional household cluster survey to determine the prevalence of filariasis infection, morbidity and mass-drug administration coverage in four townships of the Mandalay Region: Amarapura, Patheingyi, Tada-U and Wundwin. The survey included 1014 individuals from 430 randomly selected households in 24 villages. Household members one year and older were assessed for antigenaemia using immunochromatographic test cards and if positive, microfilaraemia by night-time thick blood smear. Participants 15 years and older were assessed for filariasis morbidity by ultrasound-assisted clinical examination. The overall prevalence of infection was 2.63% by antigenaemia (95% confidence interval (CI) 1.71–4.04%) and 1.03% by microfilaraemia (95%CI 0.59–1.47%). The prevalence of hydrocoele in adult males was 2.78% (95%CI 1.23–6.15%) and of lymphoedema in both genders was 0% (95%CI 0–0.45%). These results indicate the persistence of filarial infection and transmission despite six rounds of annual mass drug administration and highlight the need for further rounds as well as the implementation of morbidity management programs in the country.

## Author summary

Lymphatic filariasis (LF) is a mosquito-transmitted worm infection that causes chronic and progressive swelling of the limbs (lymphoedema) and testis (hydrocoele). Over time this swelling results in significant disfigurement and disability for sufferers. In 2000, Myanmar commenced a National Program to Eliminate LF through the annual mass drug administration (MDA) of two de-worming medications: albendazole and diethylcarbamazine to all endemic districts. However there remains little reliable information on how well the elimination program is working, and how large the burden of disease is in the country. This study assessed the prevalence of LF and participation in the MDA program in 24 villages across four townships of Mandalay Region in central Myanmar. The study found ongoing filarial infection suggesting that the six rounds of MDA had not been sufficient to stop LF transmission in the area. It also identified a substantial burden of hydrocoele but no cases of lymphoedema. These results suggest that further rounds of effective MDA are required to halt LF transmission and highlight the urgent need for morbidity management programs in the country.

## Introduction

Lymphatic filariasis (LF) remains a major cause of permanent disability in tropical and sub-tropical countries[1]. Chronic infection with mosquito-transmitted filarial worms leads to lymphatic dysfunction, resulting in progressive, irreversible swelling of the limbs, breasts or genitals. This disfigurement causes significant pain, disability, social stigma and economic sequelae for sufferers. Worldwide, the South-East Asian Region has the highest burden of LF, accounting for half of the lost disability-adjusted life years globally.[1, 2] Within the region, Myanmar is one of the worst affected countries.[3, 4]

In response, Myanmar established a National Program to Eliminate LF (NPELF) in 2000 as part of the World Health Organization’s (WHO) broader Global Program to Eliminate LF. The NPELF drew upon historical reports, national data and a 1997 WHO multi-country prevalence mapping survey to define endemic districts (the implementation unit (IU) used in the country).[5, 6] Forty-five of the 65 districts in Myanmar were classified as LF-endemic, with 47 million people at-risk (85.5% of the total population).[4, 5, 7] Baseline, pre– mass drug administration (MDA), surveys indicated high levels of antigenaemia (20–30%) in the central and western dry zones, consistent with that seen in Myanmar migrants living in Thailand.[3, 5, 6] Meanwhile, the northern, eastern and southern areas of the country were less endemic or free from filariasis.[5, 6] In Mandalay Region, the site of this study, the baseline prevalence by microfilaraemia (mf) was 5.2% (range: 0.2 to 14.7%) and by antigenaemia ranged from 2 to >25%.[5–7] The parasite *Wuchereria bancrofti* is the sole documented cause of LF in Myanmar, where it is transmitted by the mosquito *Culex quinquefasciatus*.[3, 5, 7, 8]

Under the first component of the Global Program to Eliminate LF, Myanmar began conducting annual, single-dose MDA with albendazole and diethylcarbamazine (DEC) in 2001.[4, 5] Since then, the coverage has been progressively expanded, reaching all endemic districts by 2015.[7] With the exception of six districts (three in Sagaing Region, two in Mandalay Region and one in Magway Region) that have passed Transmission Assessment Surveys (TAS), all are continuing MDA rounds.[5, 7] Whilst the WHO recommends annual MDA rounds for at least five consecutive years, several challenges resulted in two to three year intervals between rounds. At the national level, problems in the supply of DEC precluded MDA in 2005 and 2008, whilst financial and logistical issues later prevented MDA in 2012.[4, 5, 7] Additionally in the Mandalay Region, MDA did not occur in 2006 because of the reported incidence of serious adverse reactions during the preceding rounds, and in 2010 due to further DEC supply issues.[5] As a result, six non-consecutive rounds of MDA had been completed in Mandalay Region over an 11-year period from 2004 to 2014 inclusive.

Although MDA has been or is now being conducted in all endemic IUs in Myanmar, data on treatment coverage and the impact of MDA on infection prevalence have been lacking. Two papers (Win et al. 2018 and Aye et al. 2018) recently summarised NPELF data on infection prevalence and treatment coverage surveys.[5, 7] They report that whilst MDA rounds were interrupted in several districts, treatment coverage has been consistently high (60.0% to 98.5%), with significant reductions in prevalence in most areas between 2001 and 2016. In Mandalay Region, mean mf prevalence in sentinel sites has decreased from 5.0% in 2003 to 1.2% in 2014, although some districts have demonstrated persistently high levels of mf prevalence. This NPELF data provides a useful overview of Myanmar’s progress toward LF elimination, but the limitations of programmatic data are acknowledged. Reliance on repeated sampling of sentinel sites in historically high suspected prevalence areas could have over- or under estimated current prevalence and program impact given the focal distribution of LF, while programmatic data is of unknown reliability for assessing MDA coverage.[9] The program has indicated the need for in-depth studies in problem areas to independently verify Myanmar’s progress toward LF elimination.[5]

Information on the burden of LF-related morbidity in Myanmar is incomplete. A 2004 NPELF questionnaire provides the only prevalence figures from within the country.[10] The survey assessed self-reported morbidity in 280,000 individuals in Sagaing Region and identified 520 cases of lymphoedema (0.19%) and 827 cases of hydrocoele (0.59%). Such questionnaires, however, are not sensitive or specific.[11, 12] Studies of Myanmar migrants living in Thailand provide the only other indication of the disease prevalence.[13, 14] A meta-analysis of this data suggests that there is likely to be a more significant burden of hydrocoele (12.27% 95%CI 6.84–18.90) within the population.[3] Whilst these studies provide an indication of prevalence within the country, they are not representative and do not provide any information on lymphoedema. As a result, there remains no representative data on the prevalence of LF-related morbidity in Myanmar.

In light of this, a cross-sectional survey in one of the country’s priority areas was conducted. The primary objective was to determine the prevalence of LF infection and morbidity. The secondary objectives were to assess participation in the MDA program and to explore risk factors for infection, morbidity and MDA non-participation. Prevalence data and MDA participation are reported here, whilst analysis of risk factors for infection and MDA non-participation will be reported separately.

## Methods

This paper is reported according to the Strengthening the Reporting of Observational Studies in Epidemiology (STROBE) recommendations.[15]

### Study area and population

Myanmar (formerly Burma) is a lower middle-income country in Southeast Asia with a population of 52 million people.[16] The country is administratively divided into a capital territory (Naypyitaw), seven states and seven regions. These fifteen administrative areas are further divided into districts, townships, cities, towns, village tracts (groups of adjacent villages) and villages.

A cross-sectional, population-based household survey was conducted between February and March 2015 in four townships of the Mandalay Region in central Myanmar: Amarapura, Patheingyi, Tada-U and Wundwin. Townships have a typical population of between 50,000 and 250,000 people. The NPELF identified these townships as priority areas because they were historically areas of high prevalence.

**Fig 1:**
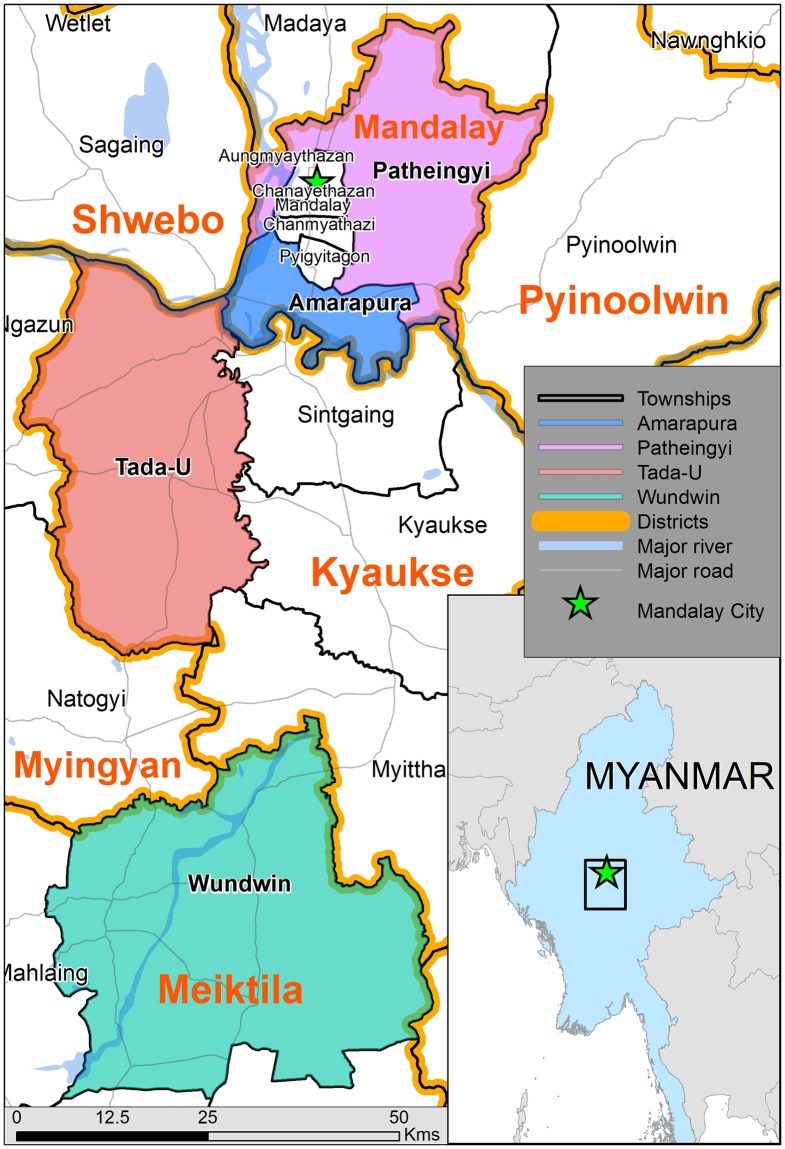
Overview of the study area including district and township boundaries (source: Geonode/MIMU 2018; Sandvik, 2018.

The townships are located directly to the north (Patheingyi) and south (Amarapura, Tada-U and Wundwin) of Mandalay, which is Myanmar’s second largest city (Fig 1). Amarapura, Patheingyi and parts of Tada-U Township lie close to the Irrawaddy River and its tributaries, whilst the Samon River passes through Wundwin. Amarapura also lies adjacent to the large Taung Tha Man Lake.

The Mandalay Region itself is situated in the low-lying and dry central plains of Myanmar. The area has two distinct seasons: the wet season from May to October (temperature range 25.9–28.6 °C) and dry season from November to April (19.1–22.8 °C).[17] The majority of rainfall occurs during the wet season (average: 140 to 197mm per month).[17]

As of 2014, the combined population of the four townships was 869,730 (Amarapura: 237,620; Patheingyi: 263,730; Tada-U: 138,620 and Wundwin: 229,760), accounting for 1.56% of the total population of Myanmar.[18] The vast majority of this population lives rurally in villages (85%), where the predominant occupation is farming.[18]

### Sampling of participants

A two-stage random cluster sampling method was used to select study participants. The sample size calculation was based on an estimated prevalence of morbidity in adults older than 15 years of 5%. Using a desired precision of 3%, an estimated design effect of two, and allowing for a non-response rate of 10% we predicted a target sample size of 880. With an average of five people per household and using a cluster size of 10 households, 234 households in 18 villages were required. To ensure the target sample size was met, we sampled 26 villages with an estimated total sample size of 260 households and 1300 individuals.

The 26 villages were selected by systematically sampling with random start from a list of all villages in the four townships.[18] The populations of villages were not available at the time of selection.

In Myanmar, villages are organised into groups of 10 households (*Sae Eain Su)*. All *Sae Eain Su* in selected villages were numbered on folded papers. The village leader then picked one from a hat. All individuals one year and older in households within the selected *Sae Eain Su* were then invited to participate. In the first two villages, one *Sae Eain Su* was selected per village, but the proportion of participants per household was lower than anticipated so two *Sae Eain Su* were then picked from each of the remaining 22 villages. To ensure maximum participation, absent households were re-visited a second time on the same day. Households that remained vacant or refused were not reselected.

### Data collection

Data collection from consenting participants included three components: an infection survey, a morbidity survey and a risk factor questionnaire.

#### Infection survey

Individuals one year and older were tested for circulating filarial antigen, signifying current infection, using the Binax Now Filariasis immunochromatographic test (ICT) cards (Alere Inc, MA, USA) on finger-prick blood samples according to manufacturer’s instructions.[19, 20] All ICT card batches were checked with positive control antigen to ensure test validity. The result was read at exactly 10 minutes after closing the card. Equivocal outcomes were re-tested.

Antigen positive individuals were referred to the LF program for treatment and re-visited in their homes for collection of blood slides between 2200 and 0200 hours by local staff (due to night-time travel restrictions for foreign investigators). Sixty μL of finger-prick blood was taken with heparinised capillary tubes and applied to duplicate microscope slides using the three-line technique.[21] The dried slides were dehaemaglobinised, fixed with methanol and stained with 2% Giemsa for 50 minutes. Two independent readers examined the slides under x100 magnification to assess for the presence and density of microfilariae and then compared their results. Microfilariae were identified to species level (*W*. *bancrofti* vs *Brugia malayi*) based upon morphologic features according to published references.[22, 23]

#### Morbidity survey

One or more medical doctors (depending on the village) and the PI (a medical student at the time of the study) trained health workers from the Ministry of Health in LF clinical examination in the field prior to data collection. This included the identification of acute filariasis manifestations (including tender lymphadenopathy, lymphangitis and cellulitis) and chronic limb manifestations (including limb circumference at defined points, the Stemmer sign and the presence of oedema and cutaneous stigmata of lymphoedema). The trained health workers then examined the limbs and lymph nodes of all participants 15 years and older for signs of acute filariasis morbidity and chronic limb lymphoedema. At least one medical doctor from Myanmar Ministry of Health (Dr Thet Wai Nwe, Dr Tint Wai or Dr Yi Yi Win), WHO (Dr San San Win) or a private GP (Dr Myint Shwe) was present for data collection in all villages. A medical doctor and the PI closely supervised examinations to ensure quality control, and conducted a clinical history and examination on all individuals with abnormalities to determine whether they were LF-related. Staging of LF-related lymphoedema was done using the Dreyer system.[24]

The PI received training in ultrasound-assisted hydrocoele examination from Professor Christopher King over one week in an LF-endemic area of Papua New Guinea in June 2014. The PI clinically examined the genitalia of participating males 15 year and over to assess for hydrocoele and scrotal lymphoedema. A medical doctor and the PI further assessed individuals identified with scrotal pathology to determine whether it was LF-related. Suspected hydrocoeles were confirmed using ultrasound (Sonosite M-Turbo, Sonosite, Washington, USA) and staged using the system outlined by Capuano et al in 2012.[25] The clinical aspects of the study were supervised by an infectious diseases physician (WJM).

#### Risk factor questionnaire

A pre-coded questionnaire was used to assess risk factors for LF infection and morbidity, as well as factors associated with participation in the MDA program. The questionnaire was translated into Myanmar language (Burmese) and pre-tested with staff of the Vector Borne Disease Control Unit. Trained health workers completed the questionnaire with the household head.

### Data analysis

Collected data was entered into an Excel database and analysed using STATA version 14 (STATA Corporation, Texas, USA). Percentage prevalence estimates with 95% confidence intervals (95%CI) were generated for infection and morbidity data. It was assumed that antigen negative individuals were also mf negative, and mf prevalence was calculated accordingly. Prevalence estimates were weighted for age and gender (using the census population distribution), weighted for sampling probability (using the number of *Sae Eain Su* and population of each village), and adjusted for clustering effect (using the *svy* command in STATA). Infection and morbidity case estimates were generated using adjusted prevalence estimates with 2014 census population data. The prevalence of infection and morbidity prevalence was classified as low, medium, high or very high as outlined previously.[3] Maps of LF prevalence and MDA participation were created with open source base-layers from Geonode/MIMU (Myanmar Information Management Unit) and the World Borders Dataset of ThematicMapping.org using ArcGIS 10.5 (ESRI, Redlands, CA).[26, 27] Univariate analyses with χ^2^ and t-tests were used to compare variables. P-values less than 0.05 were considered significant. Odds-ratios (OR) were generated using univariate logistic regression. Incomplete data were excluded from analyses.

### Ethics statement

The study was approved by The Human Research Ethics Committee, James Cook University (approval H5849) and The Ethics Review Committee on Medical Research Involving Human Subjects, Department of Medical Research, Myanmar Ministry of Health. The Ministry of Health, Regional Health Director and village leaders provided permission for this study. Written informed consent was obtained from all participants or their guardians.

## Results

### Characteristics of the study population

Fig 1 shows the location of the four townships within the Mandalay Region. Table 1 outlines the demographic characteristics of the source population and study sample. Two selected villages located in the mountains of eastern Patheingyi Township had to be excluded because of poor quality roads that are only accessible by motorcycle year-round. A total of 1014 individuals from 430 households in 24 villages participated in the study. Twenty households (4.4%) were excluded from the study: 15 were absent and five declined to participate. The median number of inhabitants per household was 4 people (interquartile range (IQR): 3, range: 1–12) with an average household monthly income of 30,000 Myanmar Kyat (18.7 USD) per person (IQR: 31,250, range: 3,000 to 375,000). The median age of participants was 36 years (IQR: 30, range: 1–86) with no significant difference in age between genders. There were significantly more females than males in the sample (63.7% vs 36.3%, p<0.001).

**Table 1:** Demographic characteristics of source population and sample.

Characteristic	Total	Township			
		Amarapura	Patheingyi	Tada-U	Wundwin
*Population*	869,730	237,620	263,730	138,620	229,760
*Number of Villages*	720	182	149	171	218
*Number of Included Villages*	24	7	4^a^	6	7
*Number of Included Households*	430	133	47	111	139
*Number of Participants*	1014	305	116	268	325
*Male (%)*	368 (36.3)	88 (28.9)	43 (37.1)	97 (36.2)	140 (43.1)
*Female (%)*	646 (63.7)	217 (71.2)	73 (62.9)	171 (63.8)	185 (56.9)
*Median Age* *(IQR*^b^, *range)*	36 (30, 1–86)	36.5 (27, 2–86)	33.5 (35.5, 1–82)	32 (31.5, 4–78)	38 (31, 2–82)

^a^Two villages excluded because of inaccessibility

^b^Interquartile range (IQR)

### Prevalence of LF infection

**Fig 2:**
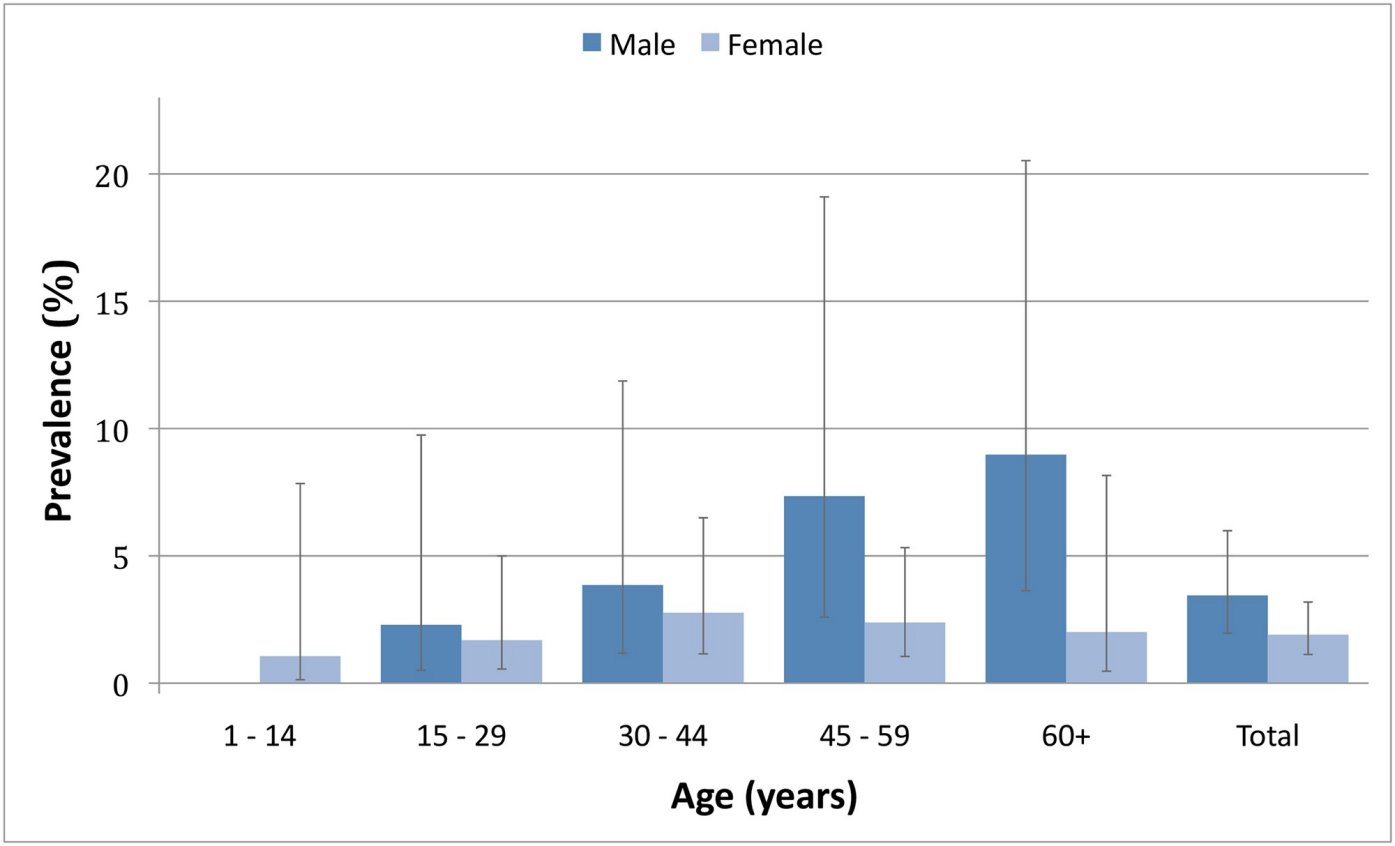
Weighted and adjusted antigenaemia prevalence by age and gender.

Table 2 outlines the prevalence of infection in the 1001 tested participants by age, gender and township. Fig 2 demonstrates the adjusted antigenaemia prevalence by age and gender. S1 Table shows crude and adjusted infection and morbidity prevalence estimates by village.

**Table 2:** Weighted and adjusted prevalence and density of LF infection^a^.

	Antigenaemia					Microfilaraemia (mf)				
	No. ICT Tests (n)	No. Positive (n)	Crude Prevalence (%) (95% CI)	Weighted and Adjusted Prevalence (%) (95% CI)	Case Estimates (n) (95% CI)	No. Slides (n)	No. Positive (n) (%)	Weighted and Adjusted Prevalence (%) (95% CI)	Case Estimates (n) (95% CI)	Weighted and Adjusted GMI (microfilariae/mL) (95% CI)^b^
**Total**	1001	46	4.60 (3.38–6.08)	2.63 (1.71–4.04)	22,874 (14,872–35,137)	41	16 (39.02)	1.03 (0.59–1.47)	8,958 (5,131–12,785)	122
**Gender**										
*Male*	365	22	6.03 (3.82–8.98)	3.45 (1.96–5.99)	14,251 (8,096–24,743)	19	8 (42.11)	1.34 (0.59–2.10)	5,535 (2,437–8,674)	67
*Female*	636	24	3.77 (2.43–5.56)	1.90 (1.13–3.19)	8,677 (5,160–14,567)	22	8 (36.36)	0.74 (0.36–1.13)	3,379 (1,644–5,160)	191
**Age**										
*<12*	145	1	0.69 (0.02–3.78)	0.56 (0.07–4.21)	1,263 (158–9,497)	1	0 (0)	0.21 (0–0.66)	474 (0–1,489)	–
*≥12*	856	45	5.26 (3.86–6.97)	2.98 (1.98–4.48)	19,196 (12,754–28,858)	40	16 (40.00)	1.16 (0.69–1.64)	7,472 (4,445–10,564)	122
**Township**										
*Amarapura*	297	33	11.11 (7.77–15.25)	6.70 (4.21–10.52)	15,921 (10,004–24,998)	30	13 (43.33)	2.62 (1.41–3.82)	6,226 (3,350–9,077)	122
*Patheingyi*	113	1	0.88 (0.02–4.83)	1.63 (0.81–3.26)	4,229 (2,136–8,598)	0	0	0.64 (0.19–1.08)	1,688 (501–2,848)	–
*Tada-U*	267	8	3.00 (1.30–5.82)	4.73 (2.43–9.01)	6,557 (3,368–12,490)	7	2 (28.57)	1.85 (0.63–3.06)	2,564 (873–4,242)	69
*Wundwin*	324	4	1.23 (0.34–3.13)	0.98 (0.31–3.08)	2,252 (712–7,077)	4	1 (25.00)	0.38 (0–0.82)	873 (0–1,884)	183

^a^Weighted for age, gender and selection probability; adjusted for survey design

^b^Geometric mean intensity (GMI) of infection (No 95%CI because of a stratum with a single sampling unit)

The overall weighted and adjusted prevalence estimate for antigenaemia was 2.63% (95% confidence interval (CI) 1.71–4.04). Using antigenaemia as a marker of active infection [28], this represents an estimated 19,637 (95%CI 12,768–30,162) individuals with LF infection within the study area. The median age of infected individuals was 46 (IQR: 23, range: 8–81 years). Of the 145 individuals born since the commencement of MDA (less than 12 years old), one was antigen positive. Six of the 1001 participants (0.60%) were re-tested because of an equivocal initial result. Of the repeat tests, four were negative and two were positive. Antigenaemia prevalence increased with age (adjusted (a) OR = 1.03 per year, 95%CI 1.01–1.05, p = 0.008) and was higher in males (aOR 2.34, 95%CI 1.03–5.36, p = 0.044) and in Amarapura Township (aOR 9.96, 95%CI 2.98–33.29, p = 0.001 using Wundwin as reference).

Microfilariae were found in 39.02% of antigen positive individuals, representing an overall adjusted mf prevalence estimate of 1.03% (95%CI 0.59–1.47%) (assuming all antigen negative persons were also mf negative). The median age of microfilaraemic individuals was 49 (IQR: 20.5, range: 27–81). Blood slide examination identified only *W*. *bancrofti*. Geometric mean intensity of infection was 122 microfilariae per mL (range: 17–2867 microfilariae per mL).

**Fig 3:**
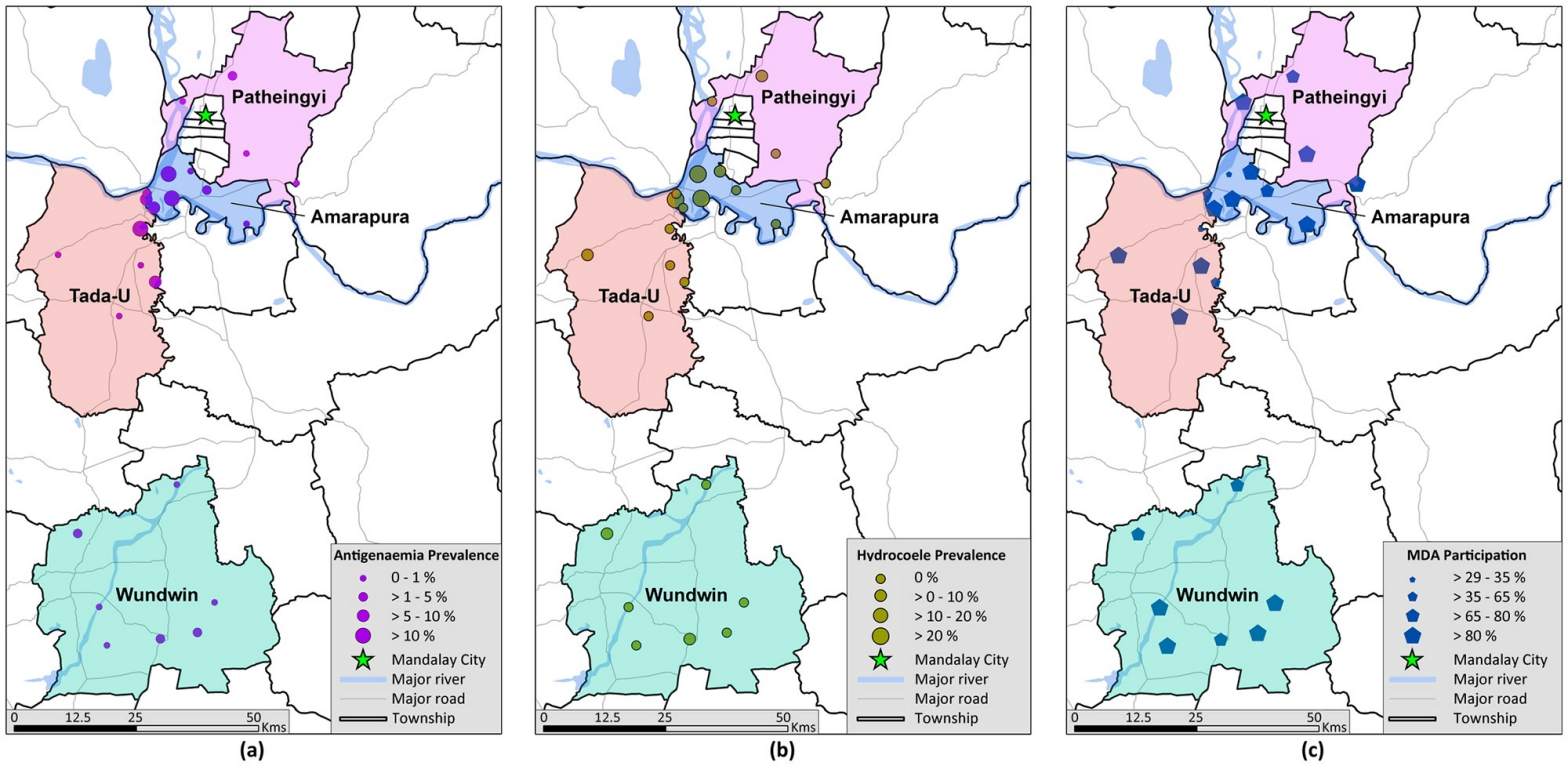
Weighted and adjusted (a) antigenaemia prevalence by ICT, (b) hydrocoele prevalence and (c) proportion of individuals who had consumed MDA medication at least once by village (source: study data; Geonode/MIMU 2018).

Fig 3 demonstrates the distribution of infection across the sampled villages. Antigen positive individuals were identified in all townships and in 12 of the 24 villages (50%). Participants with mf were found in all three townships and five of the 10 villages where thick blood films were taken. The study identified a cluster of five villages in western Amarapura and northern Tada-U townships where the mean village-level antigenaemia prevalence was 13.32% (range: 8.12–17.33%). By contrast, the mean prevalence outside the cluster was only 1.14% (range: 0.00–5.91%).

### Prevalence of LF morbidity

Table 3 shows the prevalence estimates for chronic LF morbidity and S1 Table gives a breakdown by village. Of the 824 participants (99% of those eligible) aged 15 years or above who were examined for signs of clinical LF morbidity, none were experiencing acute dermatolymphangioadenitis at the time of assessment (unadjusted 0%, 95%CI 0–0.45%).

**Table 3:** Weighted and adjusted prevalence of chronic LF morbidity^a^.

Township	Limb Lymphoedema				Hydrocoele				
	No. Examined (n)	No. positive (n)	Crude Prevalence (%) (95%CI)	Case Estimates^b^^,^^c^ (n) (95%CI)	No. Examined (n)	No. Positive (n)	Crude Prevalence (%) (95%CI)	Adjusted Prevalence (%) (95%CI)	Case Estimates^c^ (n) (95%CI)
**Total**	824	0	0 (0–0.45)	0 (0–2,899)	269	15	5.58 (3.15–9.03)	2.78 (1.23–6.15)	8,505 (3,763–18,815)
**Township**									
*Amarapura*	265	0	0 (0–1.38)	0 (0–2,429)	59	11	18.64 (9.69–30.92)	8.29 (3.71–17.47)	6,929 (3,101–14,602)
*Patheingyi*	84	0	0 (0–4.30)	0 (0–8,399)	27	1	3.70 (0.09–18.97)	3.95 (0.74–18.43)	3,664 (686–17,097)
*Tada-U*	200	0	0 (0–1.83)	0 (0–1,879)	64	1	1.56 (0.04–8.40)	0.93 (0.13–6.13)	453 (63–2,989
*Wundwin*	275	0	0 (0–1.33)	0 (0–2,263)	119	2	1.68 (0.20–5.94)	0.79 (0.17–3.56)	638 (137–2,877)

^a^Weighted for age, gender and selection probability; adjusted for survey design

^b^Based on crude prevalence,

^c^Estimates for population aged 15 years and older

Eleven cases of bilateral lower limb oedema were found, however none were clinically considered to be LF-related lymphoedema (unadjusted 0%, 95%CI 0–0.45%). As the prevalence was 0%, an adjusted prevalence estimate could not be generated.

Fifteen of the 269 males who underwent ultrasound-assisted scrotal examination (92% of those eligible) had an LF-related hydrocoele. Three cases of likely non-LF related hydrocoele were excluded: two participants stated they were present from birth, and one reported it was precipitated by trauma. After adjustment, the overall prevalence estimate for hydrocoele in males over 15 years was 2.78% (95%CI 1.23–6.15) representing approximately 8,505 cases in the study area. The median age of individuals suffering from LF-related hydrocoele was 55 (IQR: 12, range: 48–71 years). Nine of the participants with a hydrocoele (60%) were asked about surgery, with two (22%) having undergone previous hydrocoelectomy.

Higher prevalence of hydrocoele was associated with increased age (aOR 1.05 per year, 95%CI 1.03–1.08, p = 0.000) and with residing in Amarapura Township (aOR 11.11 (1.85–66.89, p = 0.011, Wundwin as reference). The presence of hydrocoele was not significantly associated with antigenaemia (p = 0.088) or mf (p = 0.076).

Fig 3 and S1 Table show the distribution of the hydrocoele burden at the village level. Cases of hydrocoele were identified across all townships and in 8 of the 24 villages (33%). Three villages in Amarapura Township (all within the ‘high infection prevalence’ cluster) demonstrated substantial prevalence of hydrocoele between 21.53 and 36.81%.

**Fig 4:**
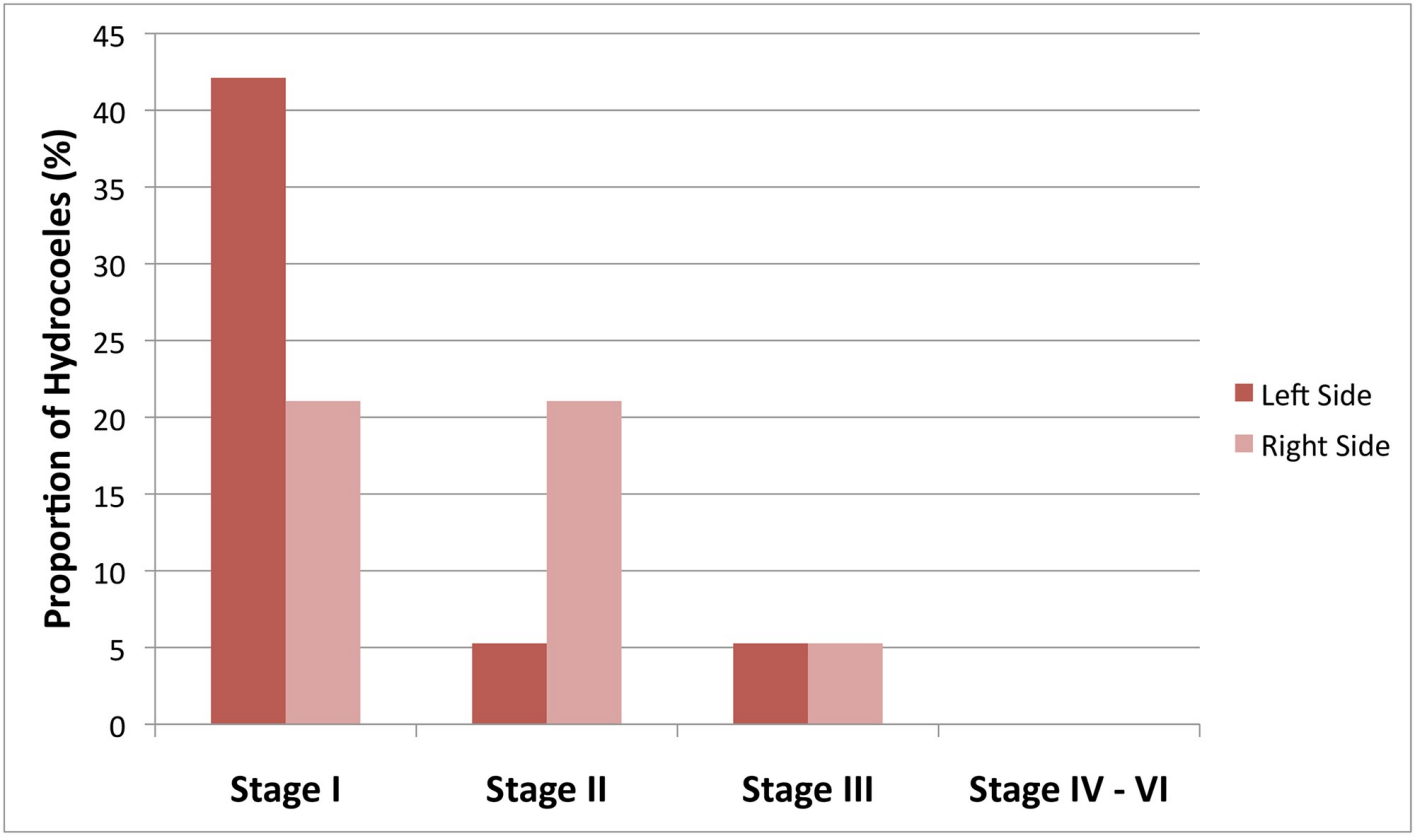
Severity of identified hydrocoele cases.

Fig 4 depicts the severity of hydrocoele cases. Eleven cases were unilateral (73%) and four bilateral (27%), representing a total of 19 hydrocoeles. Seventy-four percent were early stage (I or II). One case of bilateral stage III hydrocoele also had scrotal lymphoedema (elephantiasis). No stage IV–VI cases were observed.

### Participation in the mass drug administration program

**Fig 5:**
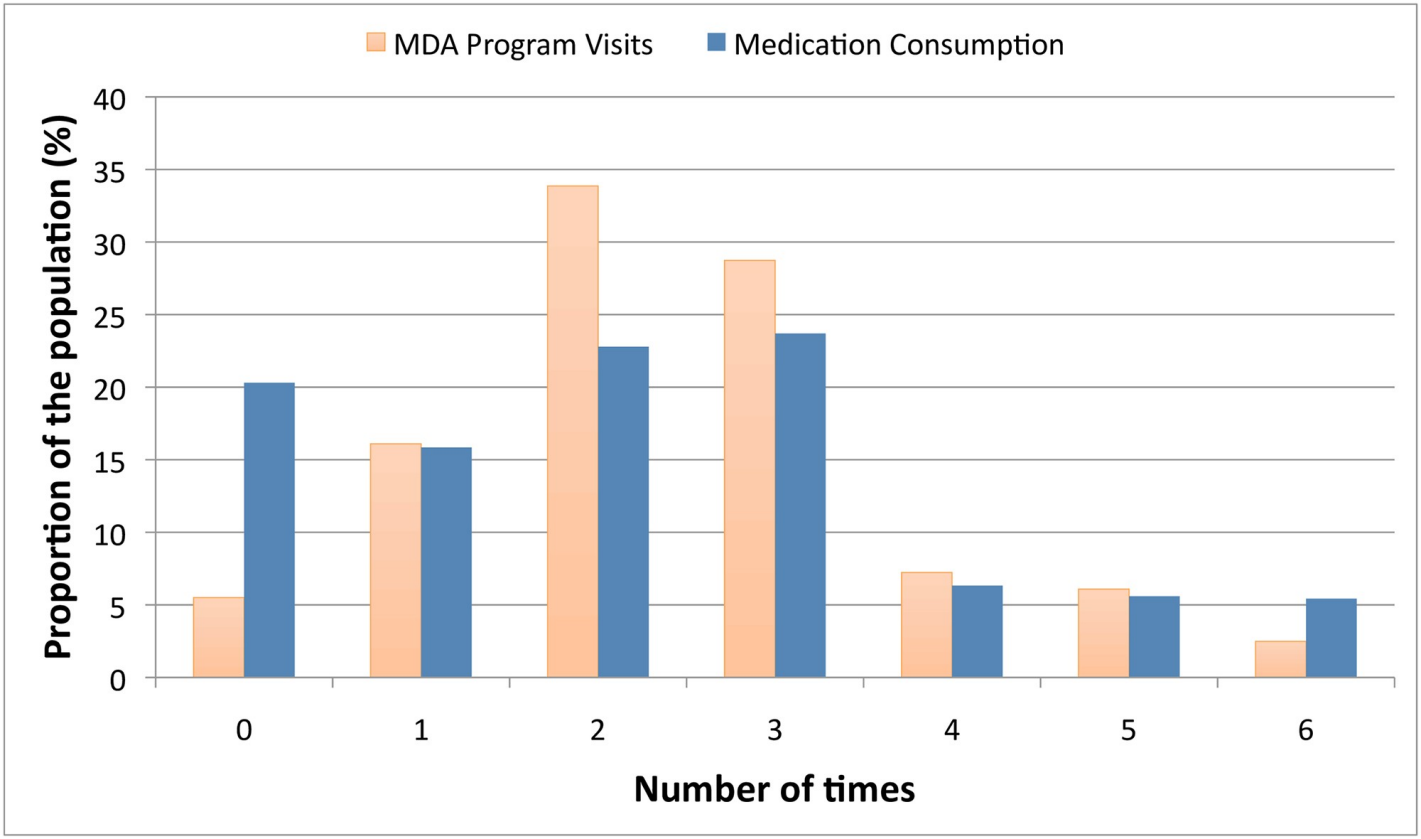
Weighted and adjusted mass drug administration coverage.

Fig 5 demonstrates the number of times participants reported being visited by the National LF program and taking medication during the MDA rounds. Ninety-five percent of households (95%CI 92.20–96.30%) reported being visited by the MDA program at least once. Of these, the mean number of visits was 2.59 (95%CI 2.44–2.74). Ninety percent of household members reported being present during the last MDA round in 2014 (95%CI 86.54–92.73%).

Eighty percent (95%CI 73.88–84.60%) of participants reported taking MDA medication at least once. Of those, the mean number of times MDA medication had been taken was 2.73 (95%CI 2.60–2.86) and 94.25% (95%CI 91.23–96.28%) had taken it in the last year. Based on this, the calculated drug coverage (i.e. proportion of the eligible population who consumed MDA medication) for the study population during the 2014 MDA was 78.76% (95%CI 72.71–83.76%).

Two hundred and twenty-nine participants (24%) reported taking MDA medication on more occasions than their household head stated they were visited by the NPELF. When those participants were excluded, the mean frequency of medication consumption reduced to 2.37. Similarly when the frequency of ingestion was capped by the number of household visits, the mean was 2.28. Alternatively, if the number of household visits was raised to match the number of times medication was ingested, the mean number of household visits increased to 2.94.

Self-reported MDA participation was lower in males (aOR: 0.62 95%CI 0.43–0.91, p = 0.017), among antigen positive individuals (aOR 0.27, 95%CI 0.11–0.66, p = 0.006) and residents of Amarapura (aOR 0.55, 95%CI 0.33–0.93, p = 0.027, with Wundwin as reference) and Tada-U (aOR 0.54, 95%CI 0.33–0.91, p = 0.022) townships. There was no association between having ever taken MDA medication and age (p = 0.739).

Fig 3 and S1 Table show the proportion of the population that had taken MDA medication at least once by village. In five villages (20.8%), less than 65% of the population had ever taken medication. Of these, three villages were in the ‘high infection prevalence’ cluster.

Ten percent (95%CI 5.08–17.41) of participants had declined to participate in taking MDA medication on at least one occasion. The main reasons given were a fear of adverse drug effects (69.72%) or a mistaken belief that hypertension was a contraindication to MDA medication (24.08%). Three percent of individuals reported adverse effects from MDA medication. The most common reported symptom was dizziness (95.65%).

## Discussion

This was the first study using representative sampling that simultaneously assessed prevalence of LF infection and morbidity in Myanmar. It estimated the prevalence of LF infection and morbidity in four townships of the Mandalay Region that have all previously completed six rounds of MDA but are regarded by the program as an area of concern. The study found a prevalence of LF infection of 2.63% by antigenaemia with evidence of ongoing transmission. The MDA rounds had not occurred in consecutive years, and self-reported MDA medication consumption was low, suggesting that MDA was likely suboptimal. The prevalence of hydrocoele in adult males was 2.78% but no cases of lymphoedema were identified in the study sample. These results have supported Myanmar’s National Elimination Program by providing accurate baseline morbidity data, highlighting the need for a morbidity management program, evaluating the effectiveness of the previous MDA rounds and identifying priority areas for future interventions.

Whilst these findings have provided crucial independent information to the National Elimination Program in Myanmar, there are some limitations. Two villages in Patheingyi Township were excluded because they could only be reached by poor quality roads on motorcycle, which was not feasible with the whole study team. Whilst it is reported that health workers do travel to these villages during MDA, the added difficulty may cause them to be missed by some rounds, resulting in persistent transmission. In excluding these villages, the study may have underestimated the infection prevalence of Patheingyi Township and overall. Secondly, government regulations restricted foreign researchers to daytime data collection when men were often away working. As a result, the study had a relatively small proportion of males considering the total sample size. Whilst adjustments were made for age and gender, this smaller sample resulted in wider confidence intervals for hydrocoele prevalence estimates. Thirdly, numerous participants reported taking MDA medication more times than their household head had stated they were visited by the NPELF. This discrepancy could be either due to an over-reporting of medication consumption (social desirability bias), an under-reporting of household visits (recall bias), individuals taking medication other than through household visits, or a combination of these factors. Despite this, when the data was adjusted to account for these discrepancies, the mean number of household visits and frequency of medication consumption still always remained less than three. Lastly, although these results were powered for the four townships, they were not representative of the entire Mandalay Region or endemic areas as a whole.

Our results indicate that MDA coverage and adherence rates have not been adequate in this study area. Whilst the program had visited all villages and most households at least once, the average number of household visits indicated that most were missed by at least half of the rounds. Household absenteeism and the declining of medication (predominantly over fears of adverse drug reactions) compounded these low coverage levels. Consequently, 20% of the population had never participated in the MDA program. The remaining individuals had, on average, participated in less than half of the six rounds These findings are consistent with modelling investigations by Aye et al. [5], which suggested that MDA in Mandalay Region was less effective than elsewhere.

In the context of these non-consecutive MDA rounds, the study demonstrated ongoing LF transmission with an overall mf prevalence greater than one percent, the presence of antigenaemia in children less than 12 years old and a cluster of villages where the mean antigenaemia prevalence was 13.32%. Despite the ongoing transmission, indirect comparison with the 1997 WHO study and data from sentinel/spot check sites suggest a progressive reduction in prevalence and infection intensity over time.[5–7]

The areas of residual infection exhibited a focal distribution (Fig 3). The greater prevalence in Amarapura and Tada-U resulted from a cluster of highly endemic villages situated near tributaries of the Irrawaddy River and the large Thaung Tha Man Lake. The baseline prevalence in these villages was likely greater than surrounding areas given the large associated hydrocoele burden. This probably relates to its proximity to both large water bodies and urban development, both of which are likely to provide extensive larval habitats for the *Culex* vectors.[29, 30] The particular persistence of infection in these areas probably also relates to the lower MDA coverage, but this requires further analysis to account for confounding factors (currently in progress).

In addition to geographic variation, the study also demonstrated higher levels of antigenaemia in males and with increasing age, both of which are well-recognised characteristics of filariasis.[31] The gender differences possibly result from increased vector exposure, decreased consumption of MDA and/or gender-related immunological susceptibility. [32, 33]

The ongoing transmission in the study area will necessitate multiple further rounds of effective MDA with attention to secure drug supply, public awareness, and improved household coverage verified by more regular independent drug coverage surveys. The implementation of MDA with triple drug therapy, including ivermectin, DEC and albendazole, could also be considered.[34] At present, this area would not quality for triple therapy because drug coverage targets have likely not consistently been met. However, given the high effectiveness of this regime and logistical and financial limitations of the NPELF, it may shorten the duration and cost of future MDA rounds and therefore LF elimination.[35]

In addition to stopping transmission, increased attention will need to be paid to the disease burden in the country. Although prevalent, the severity of identified hydrocoele cases was predominantly mild. However, since 22% of the participants with hydrocoeles had undergone a previous hydrocoelectomy, and that those without hydrocoeles were not asked about their surgical history, the true disease prevalence and severity may have been higher. Nonetheless, the adjusted estimates indicate a hydrocoele burden of 8,505 cases (95%CI 3,763–18,815) across the four townships. Given the similar endemicity patterns and population across central Myanmar, it is reasonable to infer that this disease burden may be widespread.[5, 6] Unfortunately very few cases have been reported through the public-health system.[7] To address this under-reported burden of disease, an active case-detection system and morbidity management program is urgently needed.

Fortunately, the prevalence of LF-related lymphoedema was much lower (0%, 95%CI 0–0.45%). Whilst no cases were identified amongst participants, cases were observed outside the sample and reported in 15 of the 24 villages. Of the cases of non-LF related oedema seen, all were bilateral and clinically more consistent with transudative oedema caused by cardiovascular dysfunction. It is worth noting, however, that LF-related lymphoedema may be difficult to distinguish from other types of oedema in its early stages, resulting in an underestimation of the true lymphoedema burden. The only other lymphoedema prevalence figure comes from the NPELF self-reporting questionnaire.[10] The validity of its higher prevalence estimate (0.19%, unadjusted 95%CI 0.17 to 0.20) is uncertain given the poor specificity of such questionnaires.[10, 11, 36] Whilst no cases of lymphoedema were identified in study sample, there still be as many as 2,899 cases across the four townships, underscoring the need for a morbidity management program in the country.

### Conclusion

This representative household cluster survey of LF prevalence and morbidity in four townships of the Mandalay Region, Myanmar has reported the persistent prevalence of LF infection and estimated the burden of LF-related morbidity following six rounds of MDA. It also demonstrated suboptimal treatment coverage and adherence across six rounds of MDA. Further, sequential MDA rounds with adequate coverage will be required to achieve LF elimination. With improved MDA and subsequent reductions in infection prevalence, the incidence of new hydrocoele cases should decline over time. The existing hydrocoele burden highlights the urgent need for a morbidity management program in Myanmar.

## Supporting information

S1 Table Village level LF prevalence and participation in the MDA program. ^a^Positive cases; ^b^Crude prevalence; ^c^Weighted and adjusted prevalence; ^d^Number of participants who had ever consumed MDA medication; ^e^Crude proportion; ^f^Weighted and adjusted proportion; ^g^Mean number of times MDA medication consumed in those who have taken medication before; ^h^Unable to generate 95%CI. NB: Adjustment refers to weighting for age, gender and selection probability and adjusting for survey design.
(XLSX)

S1 Checklist STROBE checklist. (PDF)
