# Supplementary material for: The prevalence of lymphatic filariasis infection and disease following six rounds of mass drug administration in Mandalay Region, Myanmar
Source: PLoS Negl Trop Dis. 2018 Nov 12;12(11):e0006944. doi: 10.1371/journal.pntd.0006944 (PMC6258426; doi:10.1371/journal.pntd.0006944)
Supplement: S1 Checklist — (PDF) [file pntd.0006944.s002.pdf]

STROBE Statement—Checklist of items that should be included in reports of ***cross-sectional studies***

	Item No	Recommendation
**Title and abstract**	1	(*a*) Indicate the study's design with a commonly used term in the title or the abstract **Abstract, paragraph 1**
		(*b*) Provide in the abstract an informative and balanced summary of what was done and what was found **Abstract, paragraph 1**
**Introduction**		
Background/rationale	2	Explain the scientific background and rationale for the investigation being reported **Introduction, paragraphs 3 – 8**
Objectives	3	State specific objectives, including any prespecified hypotheses **Introduction, paragraph 8**
**Methods**		
Study design	4	Present key elements of study design early in the paper **Methods, paragraph 11, 15 – 17**
Setting	5	Describe the setting, locations, and relevant dates, including periods of recruitment, exposure, follow-up, and data collection **Methods, paragraphs 10 – 14**
Participants	6	(*a*) Give the eligibility criteria, and the sources and methods of selection of participants **Methods, paragraphs 15 – 17**
Variables	7	Clearly define all outcomes, exposures, predictors, potential confounders, and effect modifiers. Give diagnostic criteria, if applicable **Methods, paragraphs 19 – 23**
Data sources/ measurement	8*	For each variable of interest, give sources of data and details of methods of assessment (measurement). Describe comparability of assessment methods if there is more than one group **Methods, paragraphs 19 – 23**
Bias	9	Describe any efforts to address potential sources of bias **Methods, paragraphs 19 – 23**
Study size	10	Explain how the study size was arrived at **Methods, paragraph 15**
Quantitative variables	11	Explain how quantitative variables were handled in the analyses. If applicable, describe which groupings were chosen and why **Methods, paragraph 24**
Statistical methods	12	(*a*) Describe all statistical methods, including those used to control for confounding **Methods, paragraph 24**
		(*b*) Describe any methods used to examine subgroups and interactions **Methods, paragraph 24**
		(*c*) Explain how missing data were addressed **Methods, paragraph 24**
		(*d*) If applicable, describe analytical methods taking account of sampling strategy **Methods, paragraph 24**
		(*e*) Describe any sensitivity analyses **N/A**

**Results**		
Participants	13*	(a) Report numbers of individuals at each stage of study—eg numbers potentially eligible, examined for eligibility, confirmed eligible, included in the study, completing follow-up, and analysed<br>**Results, paragraph 26**
		(b) Give reasons for non-participation at each stage<br>**Results, paragraph 26**
		(c) Consider use of a flow diagram<br>**N/A**
Descriptive data	14*	(a) Give characteristics of study participants (eg demographic, clinical, social) and information on exposures and potential confounders<br>**Results, paragraph 26 & table 1**
		(b) Indicate number of participants with missing data for each variable of interest<br>**Results, paragraphs 26 – 42 & table 2 – 3**
Outcome data	15*	Report numbers of outcome events or summary measures<br>**Results, paragraphs 26 – 42 & table 2 – 3**
Main results	16	(*a*) Give unadjusted estimates and, if applicable, confounder-adjusted estimates and their precision (eg, 95% confidence interval). Make clear which confounders were adjusted for and why they were included<br>**Results, paragraphs 26 – 42 & table 2 – 3**
		(*b*) Report category boundaries when continuous variables were categorized<br>**Results, paragraphs 26 – 42 & table 2 – 3**
		(*c*) If relevant, consider translating estimates of relative risk into absolute risk for a meaningful time period<br>**N/A**
Other analyses	17	Report other analyses done—eg analyses of subgroups and interactions, and sensitivity analyses<br>**Methods, paragraph 24 & Results, paragraphs 29 – 30**
**Discussion**		
Key results	18	Summarise key results with reference to study objectives<br>**Discussion, paragraph 43**
Limitations	19	Discuss limitations of the study, taking into account sources of potential bias or imprecision. Discuss both direction and magnitude of any potential bias<br>**Discussion, paragraph 44**
Interpretation	20	Give a cautious overall interpretation of results considering objectives, limitations, multiplicity of analyses, results from similar studies, and other relevant evidence<br>**Discussion, paragraphs 45 – 52**
Generalisability	21	Discuss the generalisability (external validity) of the study results<br>**Discussion, paragraphs 44, 49, 50, 52**
**Other information**		
Funding	22	Give the source of funding and the role of the funders for the present study and, if applicable, for the original study on which the present article is based<br>**Reported separately**

*Give information separately for exposed and unexposed groups.

Note: An Explanation and Elaboration article discusses each checklist item and gives methodological background and published examples of transparent reporting. The STROBE checklist is best used in conjunction with this article (freely available on the Web sites of PLoS Medicine at http://www.plosmedicine.org/, Annals of Internal Medicine at http://www.annals.org/, and Epidemiology at http://www.epidem.com/). Information on the STROBE Initiative is available at www.strobe-statement.org.
